# Mr. Simmons, on the Use of Tinct. Nicotianæ

**Published:** 1802-01

**Authors:** 


### *To the Editors of the Medical and Phyfical Journal.*

Gentlemen,

THOSE deranged fenfations which are incidentally confequent on gonorrhœa, are fometimes attended with inflammation, and at others are obvioufly dependant on a morbid irritability. An adequate diverfity of treatment muft therefore obtain, though after fubduing the inflammatory fymptoms, the irritability not uncommonly continues. It is here that the practitioner is frequently baffled, and after trying the ufual means without fuccefs, the fymptoms gradually of themfelves fubfide. I had an opportunity in the fpring of employing the nicotiana in this cafe, in which it anfwered fo well, that I have been induced to requeft you to publifh an account of it.

A gentlemen, while under a courfe of bougies for ftrictures, caught a frefh infection, for which he ufed a weak vitriolic injection as formerly. But when the gonorrhœa had nearly fubfided, a pain and fenfe of fulnefs in the bladder and parts contiguous fupervened, attended with dyfuria and general inflammation. For thefe were employed, bleeding both general and local, anodyne glyfters, the warm bath, and other appropriate remedies. As the inflammation abated, the gonorrhœa returned, of which the management was now configned to the free ufe of bark and mucilaginous decoctions. The ardor urinæ and other fymptoms were removed by thefe means, leaving him him molefted only by the gleet. After fome delay he grew importunate to be relieved from the gleet, and I reluctantly confented to his again ufing the injection, agreeably to his own choice. But we were foon obliged to defift, to prevent the full recurrence of the former fymptoms, feveral of which had already recurred; and probably, by this timely precaution, their feverity was mitigated, and they did not become inflammatory. He was now directed to take thirty drops of the tincture of nicotiana twice a day, in a cup full of linfeed tea, which afforded him immediate relief, and at the end of a fortnight he was free from all uneafinefs in the pelvis. The gleet continued with little diminution notwithftanding the ufe of general tonics, and after an interval of feveral weeks, he grew again difcontented. It was therefore agreed to try the injection once more, and on a threatening of the deranged fenfations to take the tincture. The firft attempt gave him confiderable alarm and obliged him to take the drops, which, however, relieved him as before, fo that they were continued in conjunction until he had obtained a cure. His general health is fince reftored by the ufe of bark and fea bathing. The injection was compofed of fix grains of the vitriol of zinc to eight ounces of diftilled water.

In the dyfuria of old people, and in other cafes of it, I have prefcribed the tinctura nicotianæ extenfively, and with advantage. Sometimes I have thought its efficacy promoted by combining it with an equal portion of the fpiritus ætheris nitrofi, but my ufual mode of exhibition has been as above. Perhaps fome affinity may fubfift between the two difeafes, at leaft I was led to employ the nicotiana in this cafe on fuch a fuppofition, and in dyfuria I can recommend it as a valuable remedy.

